# Catalytic hydrolysis of carbonyl sulphide and carbon disulphide over Fe_2_O_3_ cluster: Competitive adsorption and reaction mechanism

**DOI:** 10.1038/s41598-017-14925-5

**Published:** 2017-10-31

**Authors:** Ping Ning, Xin Song, Kai Li, Chi Wang, Lihong Tang, Xin Sun

**Affiliations:** 1 Faculty of Environmental Science and Engineering, Kunming University of Science and Technology, Kunming, 650500 PR China; 2 Faculty of Chemical Engineering, Kunming University of Science and Technology, Kunming, 650500 PR China

## Abstract

The competitive adsorption and reaction mechanism for the catalytic hydrolysis of carbonyl sulphide (COS) and carbon disulphide (CS_2_) over Fe_2_O_3_ cluster was investigated. Compared with experimental results, the theoretical study was used to further investigate the competitive adsorption and effect of H_2_S in the hydrolysis reaction of COS and CS_2_. Experimental results showed that Fe_2_O_3_ cluster enhanced the catalytic hydrolysis effect. Meanwhile, H_2_S was not conducive to the hydrolysis of COS and CS_2_. Theoretical calculations indicated that the order of competitive adsorption on Fe_2_O_3_ is as follows: H_2_O (strong) >CS_2_ (medium) >COS (weak). In the hydrolysis process, the C=S bond cleavage occurs easier than C=O bond cleavage. The hydrolysis reaction is initiated via the migration of an H-atom, which triggers C=S bond cleavage and S–H bond formation. Additionally, we find the first step of CS_2_ hydrolysis to be rate limiting. The presence of H_2_S increases the reaction energy barrier, which is not favourable for COS hydrolysis. Fe_2_O_3_ can greatly decrease the maximum energy barrier, which decreases the minimum energy required for hydrolysis, making it relatively facile to occur. In general, the theoretical results were consistent with experimental results, which proved that the theoretical study was reliable.

## Introduction

As the by-products in the industrial production, such as closed carbide furnace tail gas, carbonyl sulphide (COS) and carbon disulphide (CS_2_) corrode pipeline equipment and influence the purity of the raw material gas^1–6^. Currently, catalytic hydrolysis provided lower levels of by-products under mild reaction conditions and has thus become the most commonly used method to remove COS and CS_2_ from industrial processes^7–12^. Several previous studies showed that Fe_2_O_3_ was suitable for catalytic hydrolysis of COS and CS_2_^13–17^. In our previous study, different active components (such as Fe, Cu, Zn, Cr, Co, Ni) supported on AC were investigated for catalytic hydrolysis of COS and CS_2_^13^. The results showed that Fe_2_O_3_/AC had the highest catalytic hydrolysis performance for COS and CS_2_, yielding a 100% removal rate of CS_2_ and COS after 330 min and 240 min respectively. For Fe_2_O_3_/AC, AC played a role of adsorbent and Fe_2_O_3_ played a role of active component. Furthermore, nano-Fe_2_O_3_/AC showed higher catalytic ability for CS_2_, which prolonged 100% removal rate of CS_2_ to 480 min^18^. However, the detailed reaction mechanisms are different for different catalysts and reaction paths. For instance, Guo *et al*. and Zhang *et al*. reported the COS hydrolysis mechanism without the use of a catalyst^19,20^. The results indicated that OH and H in H_2_O first attack the C=O and C=S bonds in COS. Li *et al*. also investigated the COS hydrolysis mechanism. The results indicated that the nucleophilic additions of water across the C=O or C=S bonds of COS were competitive^21^. Additionally, the mechanism of CS_2_ hydrolysis is identical to that of COS, and COS is an intermediate the hydrolysis of CS_2_.

Currently, little research has focused on the reaction mechanism for the simultaneous removal of COS and CS_2_. Although Fe_2_O_3_ was suitable for catalytic hydrolysis of COS and CS_2_, the corresponding reaction mechanism remains unknown. Determining the detailed steps of the reaction mechanism is necessary because they can provide a theoretical foundation for the future application and development. Therefore, this work performed theoretical study to investigate the reaction mechanism and reaction routes of the simultaneous removal of COS and CS_2_ over Fe_2_O_3_ cluster. Combined with the experimental study, this theoretical study further investigated the competitive adsorption and effect of H_2_S in the hydrolysis reaction of COS and CS_2_.

## Results and Discussion

### Experimental study analysis

**Figure 1:**
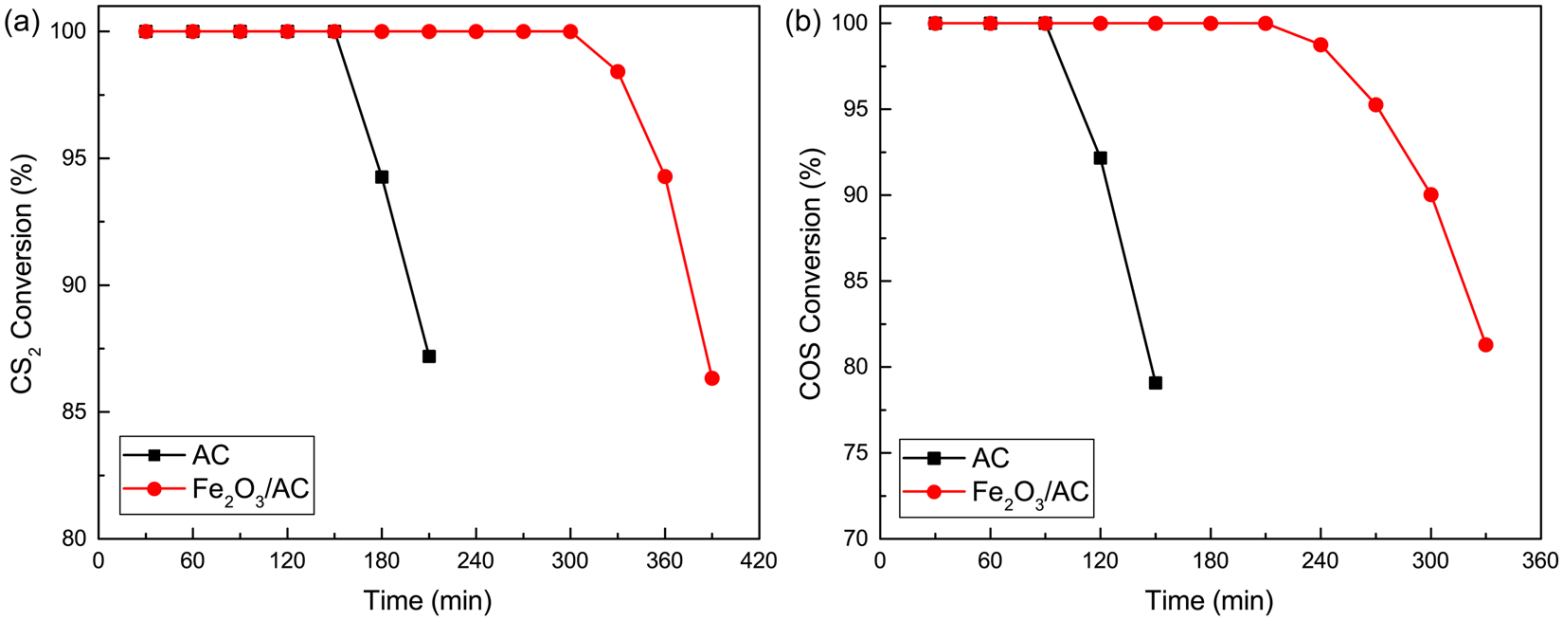
Simultaneous removal of (**a**) CS_2_ and (**b**) COS over Fe_2_O_3_/AC and pure AC (Experimental conditions: 15 ppm CS_2_; 500 ppm COS; GHSV = 10000 h^−1^; reaction temperature: 70 °C; RH = 49%; O_2_ = 0%; H_2_S = 0%).

To provide the catalytic hydrolysis effect of Fe_2_O_3_, the desulphurization experiments for COS and CS_2_ over Fe_2_O_3_/AC and pure AC were performed. The catalytic hydrolysis activities were showed in Fig. 1. From Fig. 1, the removal efficiency of COS and CS_2_ over Fe_2_O_3_/AC were higher than that over pure AC. Addition of Fe_2_O_3_ cluster prolonged the 100% COS and CS_2_ removal rate from 90 min to 210 min and 150 min to 300 min respectively. The effluent H_2_S and CO_2_ content over time was showed in Supplementary Fig. S1. As shown in Supplementary Fig. S1, the effluent H_2_S content over Fe_2_O_3_/AC firstly increased and then decreased over time. Firstly, the hydrolysis reaction rate was fast. The generation rate of H_2_S was higher than the adsorption rate, which led to the increase of H_2_S. With the increase of reaction time, H_2_S was gradually adsorbed on the surface of catalyst, which covered the active site of Fe_2_O_3_ cluster and adsorptive site of AC. As a result, it led to the deactivation of catalyst and the H_2_S content decreased. Compared with Fe_2_O_3_/AC, the effluent H_2_S content over pure AC was almost 0. Meanwhile, the CO_2_ content over Fe_2_O_3_/AC was firstly almost stable and then decreased over time. The decrease of CO_2_ content was attributed to the decrease of catalytic activity, which was caused by deactivation of catalyst. Compared with Fe_2_O_3_/AC, the effluent CO_2_ content over pure AC was almost 0. These results indicated that the removal of CS_2_ and COS over pure AC was mainly an adsorption process. Therefore, the removal of CS_2_ and COS over Fe_2_O_3_ cluster was a catalytic hydrolysis process. It indicated that Fe_2_O_3_ cluster provide the catalytic hydrolysis effect and sharply enhanced the catalytic activity.

**Figure 2:**
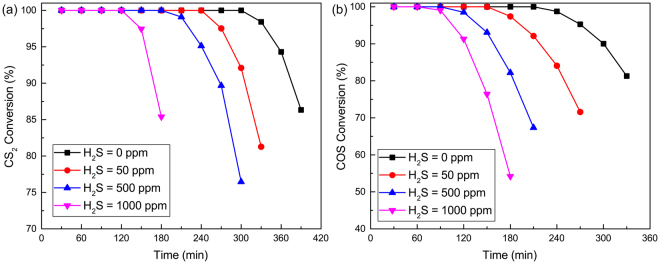
Simultaneous removal of (**a**) CS_2_ and (**b**) COS over Fe_2_O_3_/AC at different inlet H_2_S content (Experimental conditions: 15 ppm CS_2_; 500 ppm COS; GHSV = 10000 h^−1^; reaction temperature: 70 °C; RH = 49%; O_2_ = 0%).

To investigate the effect of H_2_S, the desulphurization experiments for COS and CS_2_ over Fe_2_O_3_/AC were performed. The catalytic hydrolysis activities of Fe_2_O_3_/AC at different inlet H_2_S content were shown in Fig. 2. From Fig. 2, when the content of H_2_S in the inlet was 0%, the removal efficiency of COS and CS_2_ over Fe_2_O_3_/AC was highest. Meanwhile, the removal efficiency of COS and CS_2_ decreased with increasing H_2_S content. It indicated that H_2_S is not conducive to the hydrolysis of COS and CS_2_.

To further investigate the effect of Fe_2_O_3_ and H_2_S for the catalytic hydrolysis of COS and CS_2_. The adsorption of COS and CS_2_ on Fe_2_O_3_/AC and hydrolysis reaction mechanism were studied by theoretical calculation.

### Theoretical study analysis

#### Competitive adsorption analysis

**Figure 3:**
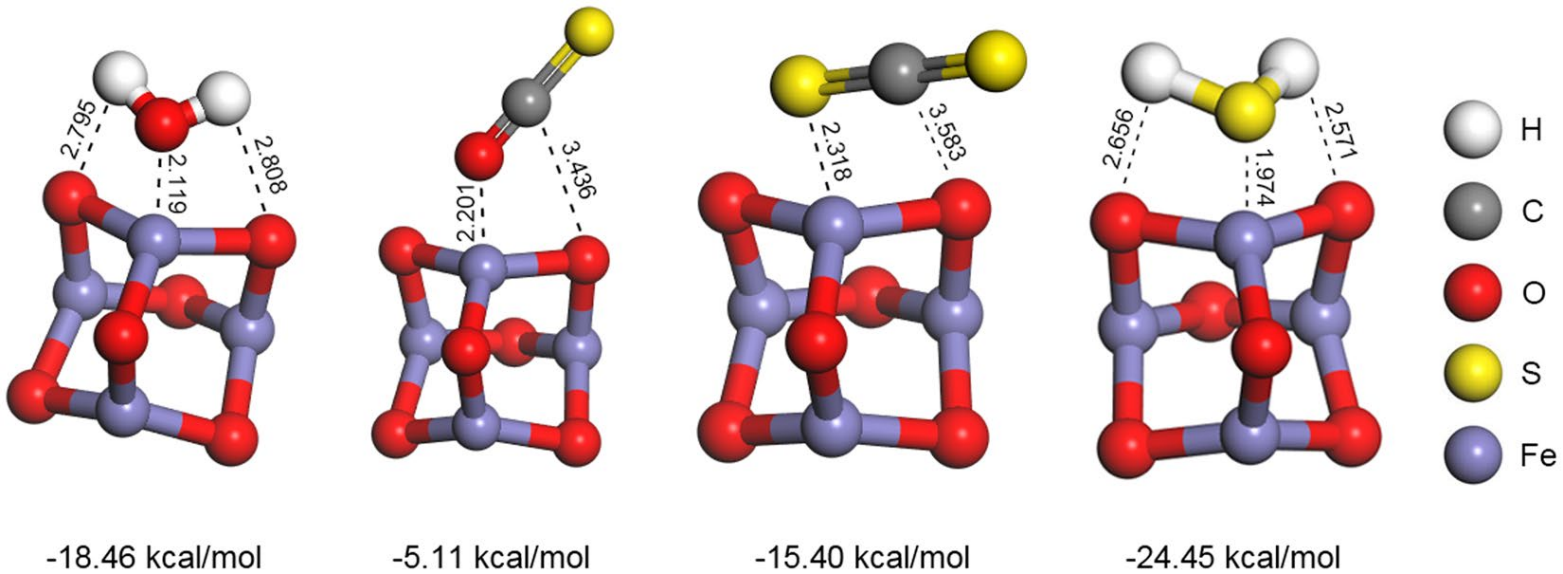
Optimized geometries and adsorption energies of H_2_S, H_2_O, COS and CS_2_ over Fe_2_O_3_ (Adsorption energy, kcal/mol).

Because the size of nano-Fe_2_O_3_ structure was less than 10 nm and it had the cluster structure, the (Fe_2_O_3_)_2_ cluster was used in the calculation study to represent the catalyst tested in experiments. To determine the relative (competitive) adsorption of H_2_S, COS, CS_2_ and H_2_O, their corresponding optimized geometries and adsorption energies have been calculated (Fig. 3). As shown in Supplementary Fig. S2, when the O atom in COS pointed to surface Fe, the absolute value of the adsorption energy was lower. It might be attributed to that the adsorption effect of C-O…Fe was more stable than C-S…Fe. As shown in Fig. 3, for reactants, the absolute value of the adsorption energy of H_2_O + Fe_2_O_3_ is the highest and that of COS + Fe_2_O_3_ is the lowest. These results indicate that H_2_O is more easily adsorbed on the Fe_2_O_3_ cluster than COS and CS_2_, which suggests that H_2_O firstly adsorbs on the catalyst and then reacts with COS and CS_2_. Meanwhile, the absolute value of the adsorption energy of CS_2_ + Fe_2_O_3_ is the higher than that of COS + Fe_2_O_3_, which suggests that the adsorption of CS_2_ on the surface of the catalyst occurs first. For the product H_2_S, H_2_S + Fe_2_O_3_ has higher absolute value of the adsorption energy than CS_2_, COS and H_2_O. It indicated that H_2_S will be firstly adsorbed on the Fe_2_O_3_, which will gradually decrease the catalytic hydrolysis activity of catalyst.

#### Reaction mechanism of COS hydrolysis over Fe_2_O_3_ cluster

**Figure 4:**
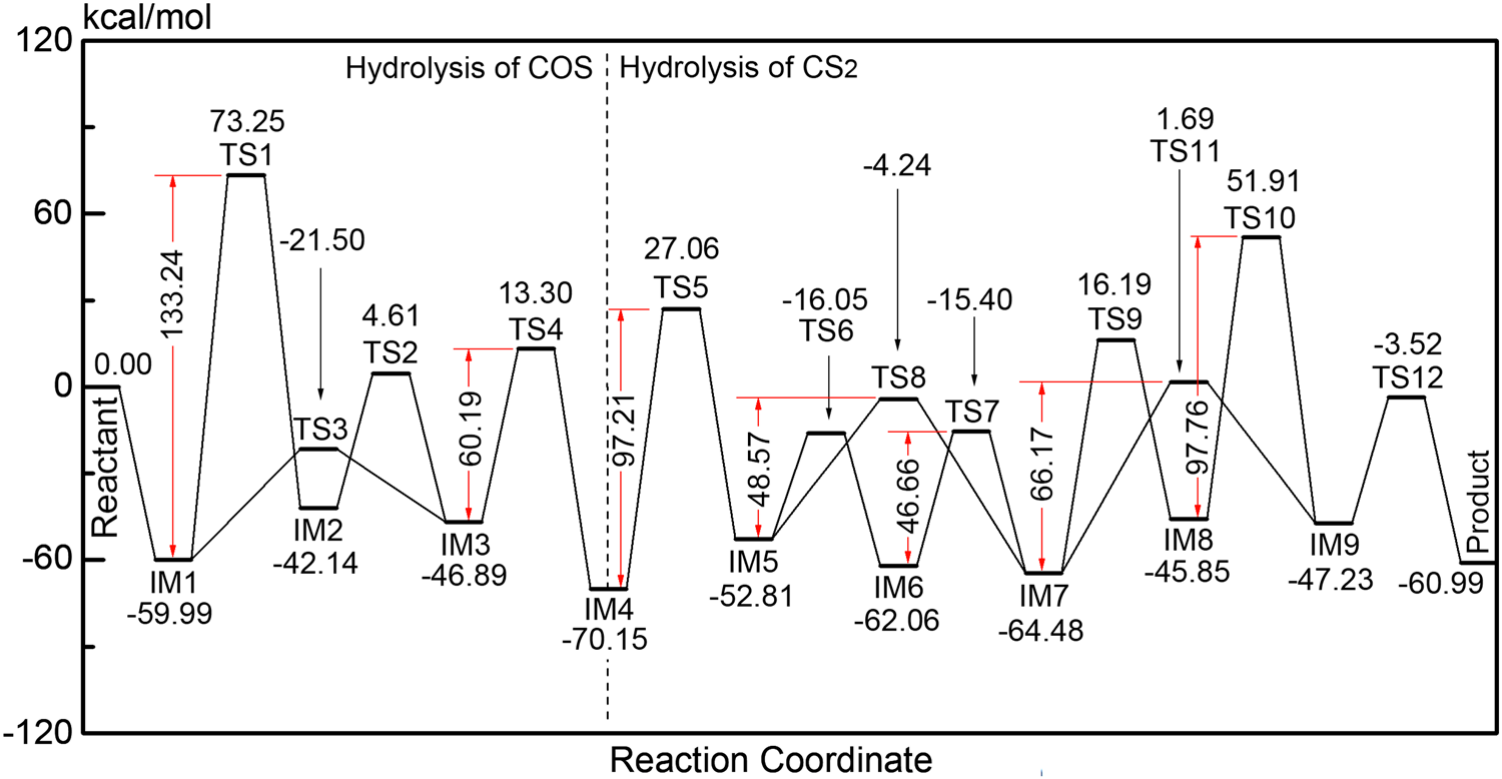
Reaction channel and potential energy surface for the hydrolysis of COS and CS_2_ The optimized geometries for the hydrolysis of COS are presented in Fig. 5. The imaginary frequencies of the transition states are given in Supplementary Table S1.

The reaction channels for the hydrolysis of COS and CS_2_ are given in Fig. 4. The bond-lengths of transition states, reaction energies and energy barriers of various reaction steps were showed in Supplementary Table S1, Supplementary Table S2, Supplementary Table S3 and Supplementary Table S4. As seen in Fig. 4 and Supplementary Table S4, the energy barrier of COS hydrolysis (60.19 kcal/mol) over Fe_2_O_3_ is lower than that of CS_2_ hydrolysis (97.21 kcal/mol), suggesting that the hydrolysis of COS occurs more easily than the hydrolysis of CS_2_. Therefore, the hydrolysis of COS occurs preferentially for the simultaneous removal of COS and CS_2_.

(1) C-O channel for COS hydrolysis.

**Figure 5:**
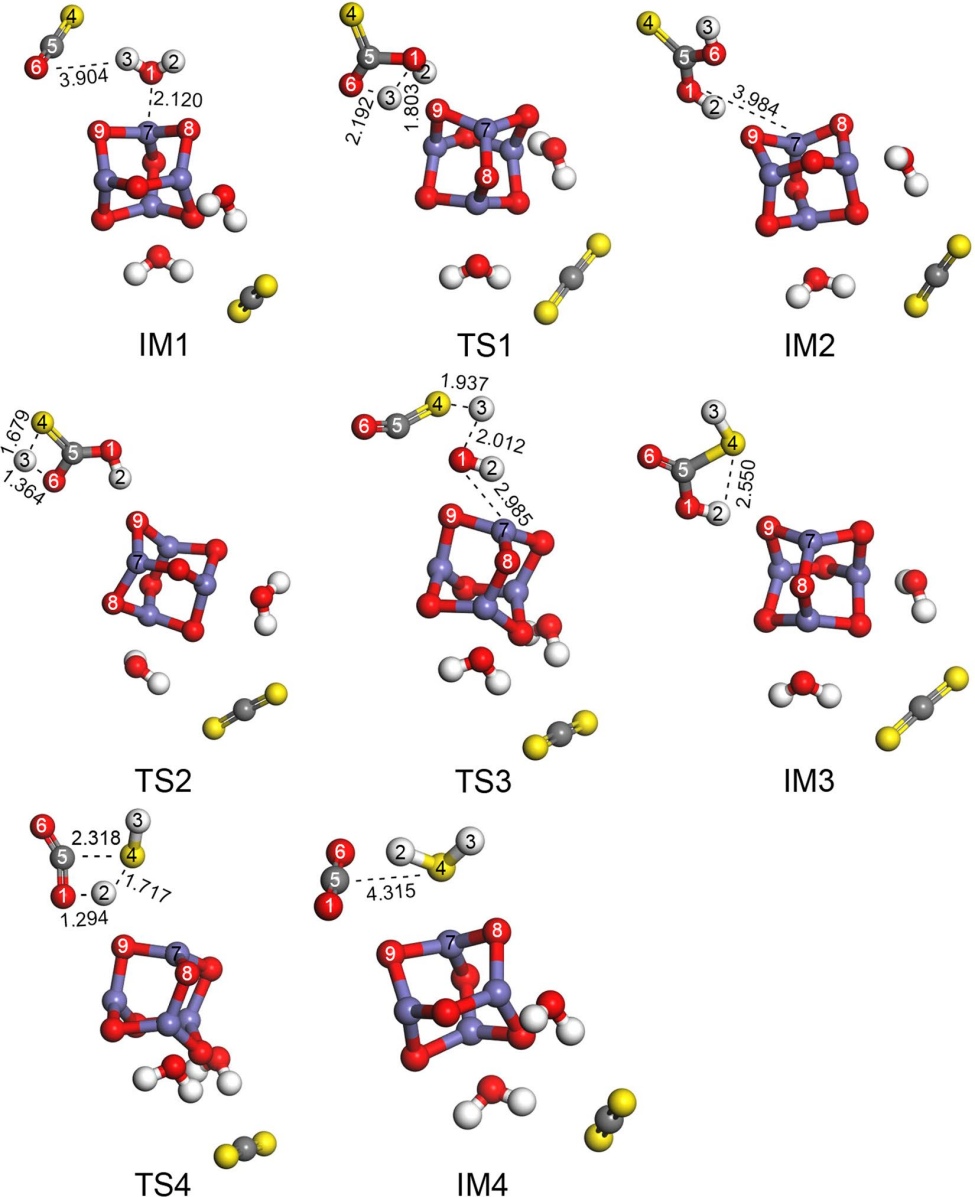
Optimized geometries (of IM and TS) for COS hydrolysis.

In reaction channel I (i.e., Reactant → IM1 → TS1 → IM2 → TS2 → IM3 → TS4 → IM4), H_2_O first adsorbs on the surface of Fe_2_O_3_, forming IM1. The C5–O1 bond length decreases and the H3–O1 bond breaks. At the same time, the C5–O1 and H3–O6 bonds are formed with the change from a C5=O6 double bond to a C5–O6 single bond to generate IM2 via TS1 (with just one imaginary frequency of –906.47 cm^–1^). As seen in Fig. 5 and Supplementary Table S1, H3 moves from its position in IM1 to O6 in IM2 with an energy barrier of 133.24 kcal/mol. In effect, H3 and H2–O1 in H_2_O attack the O6 and C5 atoms in COS, respectively.

The H3–O6 bond length then becomes longer as the H3–S4 bond length becomes shorter. Meanwhile, the H3–O6 bond breaks and the C5=O6 bond forms from the C5–O6 bond. Then, H3 migrated from the H3–O6 bond in IM2 to the H3–S4 bond in IM3. As the result, IM3 is generated via TS2 (with just one imaginary frequency of –1528.43 cm^−1^) with an energy barrier of 46.75 kcal/mol. For TS2, Fe_2_O_3_ had an adsorption effect on O9 and H2, which led to that the migration of H3 from O6 to S4 was easier than H2 from O1 to S4.

Concomitantly, the bond lengths of H2–O1 and C5–S4 become longer and the H2–S4 bond length becomes shorter. Additionally, the H2–O1 and C5–S4 bonds become broken, the H2–S4 bond forms, and the C5=O1 bond forms from the C5–O1 bond. Subsequently, the products (IM4) are formed via TS4 (with just one imaginary frequency of –1643.95 cm^–1^) with an energy barrier of 60.19 kcal/mol. H2 migrated from the H2–O1 bond in IM3 to the H2–S4 bond in IM4.

(2) C-S channel for COS hydrolysis

In reaction channel II (i.e., Reactant → IM1 → TS3 → IM3 → TS4 → IM4), H_2_O first adsorbs on the surface of Fe_2_O_3_ to generate IM1. Then, the C5–O1 bond length decreases and the H3–O1 bond breaks. At the same time, the C5–O1 and H3–S4 bonds form with the change from a C5=S4 double bond to a C5–S4 single bond, forming IM3 via TS3 (with just one imaginary frequency of −755.33 cm^–1^). H3 moves from its position in IM1 to S4 in IM3 with an energy barrier of 38.49 kcal/mol. More specifically, H3 and H2–O1 in H_2_O attack the S4 and C5 atoms in COS, respectively. For TS3, the adsorption effect of Fe7 and O1-H2 led to that the migration of H3 from O1 to H4 was easier than H2 from O1 to S4. The subsequent steps are identical to those in reaction channel I (i.e., IM3 → TS4 → IM4).

On the basis of these results, it is clear that the migration of H3 from the H3–O1 bond in IM1 to the H3–S4 bond in IM3 is easier than the migration of H3 from H3–O1 bond to the H3–O6 bond. Thus, the H–S bond forms more easily than the H–O bond, and reaction channel II is likely to occur more readily than reaction channel I.

#### Reaction mechanism of CS2 hydrolysis over Fe2O3 cluster

**Figure 6:**
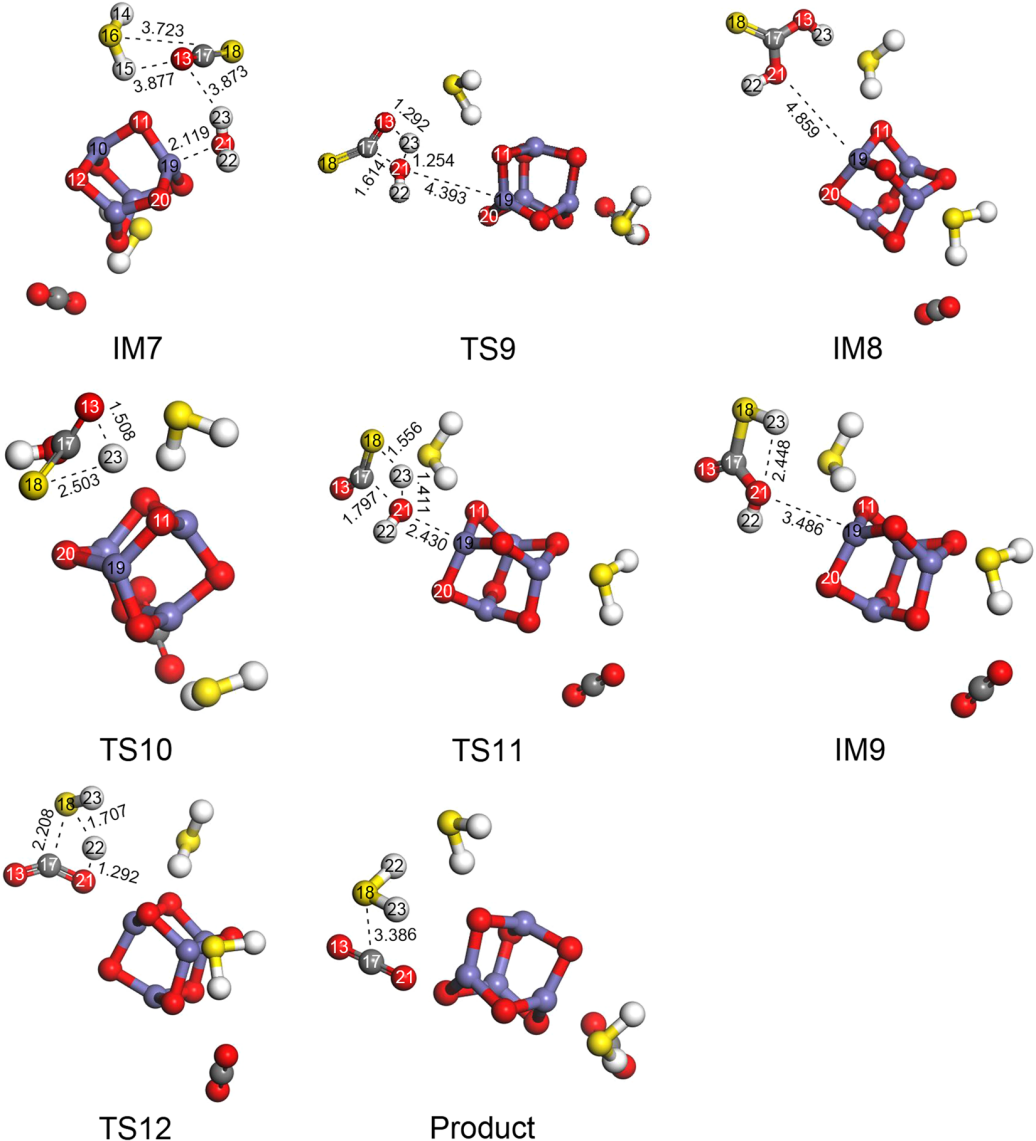
Optimized geometries (of IM and TS) for step 1 of CS_2_ hydrolysis.

**Figure 7:**
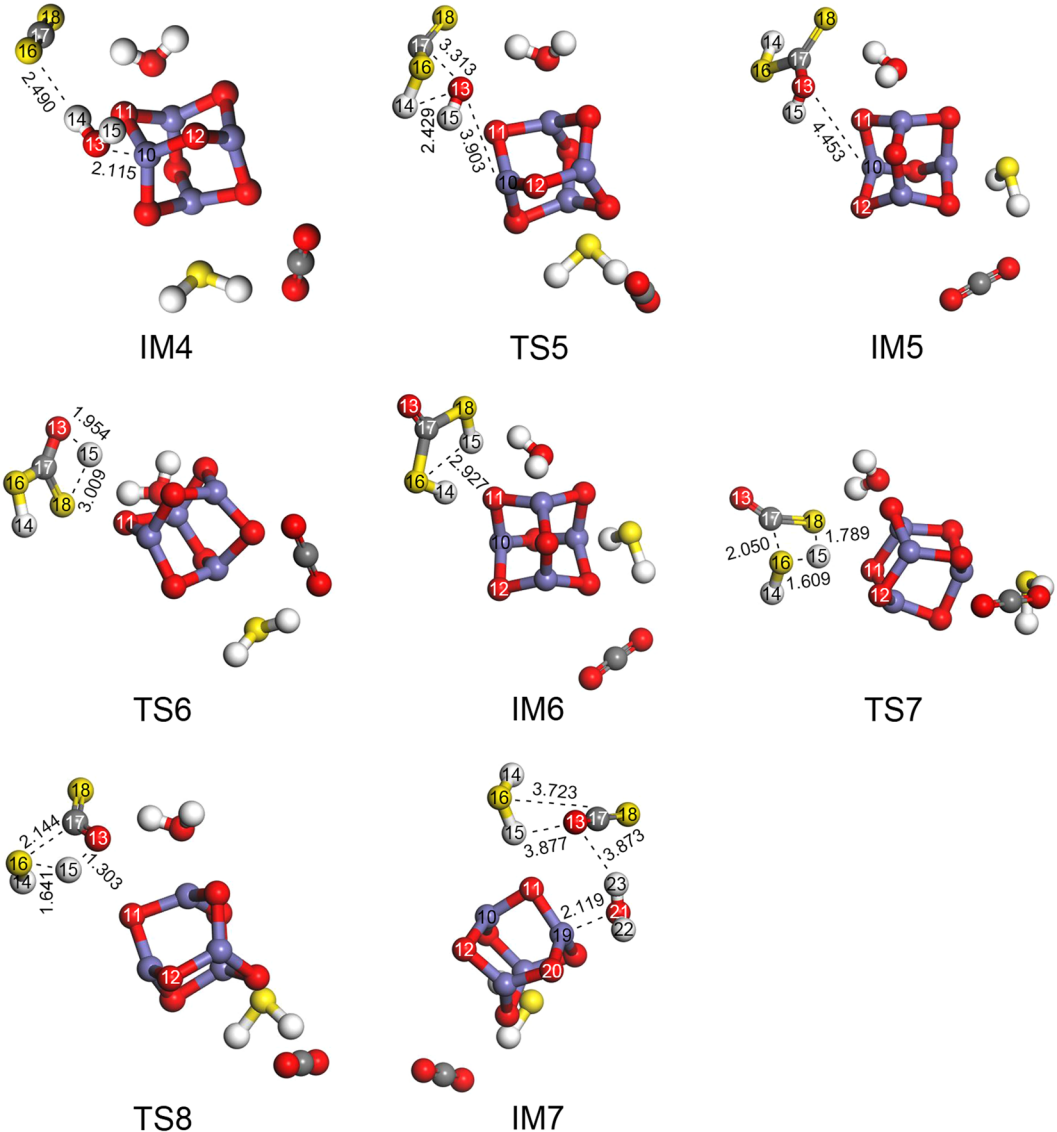
Optimized geometries (of IM and TS) for step 2 of CS_2_ hydrolysis.

As seen in Fig. 4, the hydrolysis of CS_2_ can be divided into two steps: CS_2_ → COS and COS → H_2_S. The optimized geometries for the step 1 of hydrolysis of CS_2_ are presented in Fig. 6, and the imaginary frequencies of the transition states for COS hydrolysis are shown in Supplementary Table S2. The optimized geometries for the step 2 of hydrolysis of CS_2_ are presented in Fig. 7, and the imaginary frequencies of the transition states for COS hydrolysis are shown in Supplementary Table S3.

(1) Double H-S channel for step 1 of CS_2_ hydrolysis.

In reaction channel III (i.e., the first step of CS_2_ hydrolysis: IM4 → TS5 → IM5 → TS6 → IM6 → TS7 → IM7), the C17–O13 bond length decreases and the H14–O13 bond breaks. As a result, the C17–O13 and H14–S16 bonds form with the change from a C17=S16 double bond to a C17–S16 single bond to generate IM5 via TS5, which has only one imaginary frequency of −703.92 cm^−1^. As seen in Fig. 6 and Supplementary Table S2, H14 moves from its position in IM4 to S16 in IM5 with an energy barrier of 97.21 kcal/mol. Thus, H14 and H14–O13 in H_2_O attack the S16 and C17 atoms in CS_2_, respectively.

Consequently, the H15–O13 bond length becomes longer and the H15–S18 bond length becomes smaller. Meanwhile, the H15–O13 bond breaks and the C17=O13 bond forms from the C17–O13 bond. As a result, IM6 is generated via TS6 (with just one imaginary frequency of −1045.81 cm^−1^) with an energy barrier of 36.76 kcal/mol. H15 migrated from the H15–O13 bond in IM5 to the H15–S18 bond in IM6.

Concomitantly, the H15–S18 and C17–S16 bond lengths become longer while the H15–S16 bond length becomes smaller. Additionally, the H15–S18 and C17–S16 bonds break, the H15–S16 bond forms, and the C17=S18 bond is generated from the C17–S18 bond. Subsequently, IM7 is formed via TS7 (with just one imaginary frequency of −1019.70 cm^−1^) with an energy barrier of 46.66 kcal/mol. H15 migrated from the H15–S18 bond in IM6 to the H15–S16 bond in IM7.

(2) Single H-S channel for step 1 of CS_2_ hydrolysis.

In reaction channel IV (i.e., the first step of CS_2_ hydrolysis: IM4 → TS5 → IM5 → TS8 → IM7), IM5 is formed via TS5 causing the H15–O13 bond length to become longer and the H15–S16 bond length to become shorter. Meanwhile, the H15–O13 bond breaks and the C17=O13 bond forms from the C17–O13 bond. These changes lead to IM7, which is generated via TS8 (with just one imaginary frequency of −1546.07 cm^−1^) with an energy barrier of 48.57 kcal/mol. H15 migrated from the H15–O13 bond in IM5 to the H15–S16 bond in IM7.

Based on these results, the migration of H15 from the H15–O13 bond to the H15–O18 bond in IM6 occurs more easily than the migration of H15 from the H15–O13 bond in IM5 to the H15–S16 bond in IM7. Overall, these results suggest that the H atom more easily forms an H–S bond with the C=S bond than the C–S bond and that reaction channel III occurs more easily than reaction channel IV.

(3) C-O channel for step 2 of CS_2_ hydrolysis.

As seen in Fig. 7, in reaction channel V (i.e., the second step of CS_2_ hydrolysis: IM7 → TS9 → IM8 → TS10 → IM9 → TS12 → Product), the C17–O21 bond length decreases and the H23–O21 bond breaks. At the same time, the C17–O21 and H23–O13 bonds are formed with the change from C17=O13 to C17–O13, forming IM8 via TS9 (with just one imaginary frequency of −1624.81 cm^−1^). As seen in Fig. 7 and Supplementary Table S3, H23 moves from its position in IM7 to O13 in IM8 with an energy barrier of 80.67 kcal/mol, and H23 and H23–O21 in H_2_O attack the O13 and C17 atoms in COS, respectively.

The H23–O13 bond length then becomes longer while the H23–S18 bond length becomes shorter. Meanwhile, the H23–O13 bond breaks and the C17=O13 bond forms from the C17–O13 bond. Then, H23 migrated from the H23–O13 bond in IM8 to the H23–S18 bond in IM9. As the result, IM9 is generated via TS10 (with just one imaginary frequency of −1325.24 cm^−1^) with an energy barrier of 97.76 kcal/mol.

Concomitantly, the H22–O21 and S18–C17 bond lengths become longer while the H22–S18 bond length becomes shorter. Additionally, the H22–O21 and C17–S18 bonds break, the H22–S18 bond forms, and the C17=O21 bond forms from the C17–O21 bond. Subsequently, the products are formed via TS12 (with just one imaginary frequency of −1495.37 cm^−1^) with an energy barrier of 43.71 kcal/mol. H22 migrated from the H22–O21 bond in IM9 to the H22–S18 bond in the product.

(4) C-S channel for step 2 of CS_2_ hydrolysis.

In reaction channel VI (i.e., the second step of CS_2_ hydrolysis: IM7 → TS11 → IM9 → TS12 → Product), the C17–O21 bond length decreases and the H23–O21 bond breaks. At the same time, the C17–O21 and H23–O18 bonds are formed with the change from C17=S18 to C17–S18, forming IM9 via TS11 (with just one imaginary frequency of −1172.61 cm^−1^). As seen, H23 moves from its position in IM7 to S18 in IM9 with an energy barrier of 66.17 kcal/mol, and H23 and H22–O21 in H_2_O attack the S18 and C17 atoms in COS, respectively. The subsequent steps are then identical to those in reaction channel V (IM9 → TS12 → Product).

Overall, it is clear that the migration of H23 from the H23–O21 bond in IM7 to the H23–S18 bond in IM9 occurs more easily than the migration of H23 from the H23–O21 bond to the H23–O13 bond. The results also indicate that the H–S bond forms more easily than the H–O bond, which agrees with the above results. Therefore, reaction channel VI is likely to occur more easily than reaction channel V.

### Effect of H_2_S and Fe_2_O_3_ on hydrolysis of CS_2_ and COS

According to the above results, the order of the competitive adsorption on Fe_2_O_3_ is as follows: H_2_O (strong) > CS_2_ (medium) > COS (weak). H_2_S does not change the priority of the migration of the H atom in H_2_O from the H–O bond to the H–S bond. In the hydrolysis, the C=S bond cleavage occurs easier than C=O bond cleavage. The presence of H_2_S increases the maximum energy barrier for COS hydrolysis (from 60.19 kcal/mol to 66.17 kcal/mol), which was attributed to the competitive adsorption effect. As the result, it covered the active sites and was not favourable for COS hydrolysis. Meanwhile, the first step of CS_2_ hydrolysis has a higher energy barrier than the second step, which indicates that the first step is rate-limiting. The energy barriers of gas-phase reaction were showed in Supplementary Table S5. Compared with gas-phase reaction without catalyst, all the maximum energy barriers of surface reaction were lower. The addition of Fe_2_O_3_ can greatly decrease the maximum energy barrier (159.74 kcal/mol and 182.71 kcal/mol for COS and CS_2_, respectively), which decreases the minimum energy required for the hydrolysis reaction. It was attributed to the adsorption effect between Fe_2_O_3_ and H_2_O, and H migration. From the experimental results, nano-Fe_2_O_3_ cluster promoted the catalytic hydrolysis reaction of CS_2_ and COS. It might be attributed to the interaction effect Fe_2_O_3_ and COS/CS_2_, and high catalytic hydrolysis activity of Fe_2_O_3_. As a product, H_2_S decreased the removal efficiency of COS and CS_2_ when it was on the surface of catalyst. It indicated that H_2_S was not conducive to the hydrolysis of COS and CS_2_. The results demonstrate that Fe_2_O_3_ is a good catalyst for the hydrolysis of COS and CS_2_. In general, theoretical results were consistent with experimental results, which proved that the theoretical study was reliable. Combined with theoretical and experimental results, it can be found that the influence mechanism of Fe_2_O_3_ and H_2_S for catalytic hydrolysis of COS and CS_2_.

## Conclusions

The competitive adsorption and reaction mechanism for the catalytic hydrolysis reaction mechanism of carbonyl sulphide (COS) and carbon disulphide (CS_2_) over Fe_2_O_3_ cluster was investigated in this work. The experimental results showed that Fe_2_O_3_ cluster enhanced the catalytic hydrolysis effect. Meanwhile, H_2_S was not conducive to the hydrolysis of COS and CS_2_. On the basis of the calculation results, the order of competitive adsorption on Fe_2_O_3_ is: H_2_O (strong) > CS_2_ (medium) > COS (weak). In the hydrolysis process, the C=S bond cleavage occurs easier than C=O bond cleavage. The presence of H_2_S increases the reaction energy barrier, which is not favourable for COS hydrolysis. Meanwhile, Fe_2_O_3_ can greatly decrease the maximum energy barrier, which decreases the minimum energy required for hydrolysis, making it relatively facile to occur. Combined with theoretical and experimental results, it can be found that the influence mechanism of Fe_2_O_3_ and H_2_S for catalytic hydrolysis of COS and CS_2_.

## Experimental and Theoretical Methods

### Experimental Methods

To investigate the effect of nano-Fe_2_O_3_ cluster for catalytic hydrolysis of COS and CS_2_, AC (activated carbon) and nano-Fe_2_O_3_ cluster/AC were used in this work. Nano-Fe_2_O_3_ cluster was synthesized by hydro-thermal method^22^. 4.40 g FeCl_3_ and 4.0 g urea were added into 100 mL distilled water. 50 mL NH_3_·H_2_O was added dropwise into the solution with continuous magnetic stirring. The solution was poured into a Teflon-lined container and then it was heated at 150 °C for 12 h. The solution was cooled under room temperature. The obtained precipitates were washed by ethanol and distilled water for three times. Finally, the precipitates were calcinated at 450 °C for 3 h. The size of nano-Fe_2_O_3_ structure was less than 10 nm and it had the cluster structure. Nano-Fe_2_O_3_ cluster/AC (activated carbon) was synthesized by dipping method according to our previous study^13,14^. 5% mass content of nano-Fe_2_O_3_ cluster and AC were added into 50 mL distilled water. The solution was placed in an ultrasonic bath for 30 min. Finally, the solution was dried at 120 °C for 20 h and nano-Fe_2_O_3_ cluster/AC was prepared.

The desulphurization tests were performed in a fixed–bed quartz reactor under atmospheric pressure^13,14^. CS_2_ and COS from the gas cylinder (0.3% CS_2_ in N_2_; 1% COS in N_2_) were diluted with N_2_ (99.99%) to the required concentration (CS_2_: 20 ppm; COS: 500 ppm). The overall gas hourly space velocity (GHSV) was 10000 h^–1^. Water was obtained from a saturator system, which set the temperature and corresponding relative humidity (RH) to 5 °C and 49%, respectively. The temperature of the reactor was maintained at 70 °C using a water bath. The CS_2_, COS and H_2_S (hydrolysis product) concentration of the gas feed and effluent from the reactor were analysed using a HC–6 sulphur phosphorus microscale analyser. The removal rate of CS_2_ and COS were determined by calculating the inlet and outlet concentration of CS_2_ and COS, and it was shown in Eq. 1:

1 $${{\rm{CS}}}_{{\rm{2}}}({\rm{COS}}){\rm{conversion}}\,( \% )=\frac{{{\rm{CS}}}_{{\rm{2}}}{({\rm{COS}})}_{{\rm{in}}}-{{\rm{CS}}}_{{\rm{2}}}{({\rm{COS}})}_{{\rm{out}}}}{{{\rm{CS}}}_{{\rm{2}}}{({\rm{COS}})}_{{\rm{in}}}}\times 100$$

### Theoretical Methods

All calculations in this work were performed using Dmol^3^ in the Material Studio software package^23^. The molecular geometries of the reactants, transition states (TS), intermediate complexes (IM), and products were calculated and optimized using the GGA/PBE method from density functional theory (DFT)^24,25^. A density functional semi-core pseudopotential method was used for the core electrons of Fe, and the all-electron method was used for the core electrons of H, C, O and S. A double-numeric quality basis set with polarization functions (i.e., DNP, version 3.5) was used^25–27^. The tolerances of the SCF, energy, gradient and displacement convergence were 1.0 × 10^–6^ hartree (Ha), 1.0 × 10^–5^ Ha, 2.0 × 10^–3^ Ha/Å and 5.0 × 10^–3^ Å, respectively. All calculations using spin-polarized set were performed considering the antiferromagnetic properties. In previous study, electronic self-interaction error played an important role in DFT^28,29^. Therefore, the electron self-interaction error had been consideration in this work. Self-consistent field convergence was declared when at least two of the above criteria were satisfied. Electronic energies and zero point vibration energies (ZPVE) were calculated at the same level of theory. Linear synchronous transit/quadratic synchronous transit/conjugate gradient (LST/QST/CG) calculations were used to ensure that all of the transition states connected to the intended reactants and products. Transition states were identified by the presence of a single imaginary frequency, which corresponded to the reaction mode. The adsorption energy was defined as Eq. 2. E_(adsorbate+cluster)_ is the total energy of adsorbate/cluster system after gas molecule being adsorbed on Fe_2_O_3_ cluster. E_adsorbate_ is the single-point energy of gas molecule and E_cluster_ is the single-point energy of Fe_2_O_3_ cluster.

2 $${{\rm{E}}}_{{\rm{ads}}}={{\rm{E}}}_{({\rm{adsorbate}}+{\rm{cluster}})}-{{\rm{E}}}_{{\rm{adsorbate}}}-{{\rm{E}}}_{{\rm{cluster}}}$$

## Electronic supplementary material

Supplementary materials
