# Supplementary material for: Catalytic hydrolysis of carbonyl sulphide and carbon disulphide over Fe2O3 cluster: Competitive adsorption and reaction mechanism
Source: Sci Rep. 2017 Oct 31;7:14452. doi: 10.1038/s41598-017-14925-5 (PMC5663713; doi:10.1038/s41598-017-14925-5)
Supplement: Supplementary file 1 — Supplementary materials [file 41598_2017_14925_MOESM1_ESM.pdf]

Catalytic hydrolysis of carbonyl sulphide and carbon disulphide over Fe_2O_3 cluster: Competitive adsorption and reaction mechanism

Ping Ning[a,†], Xin Song[a,†], Kai Li[a,*] Chi Wang[b], Lihong Tang[a], Xin Sun[a]

[a] *Faculty of Environmental Science and Engineering, Kunming University of Science and Technology, Kunming 650500, PR China*
[b] *Faculty of Chemical Engineering, Kunming University of Science and Technology, Kunming 650500, PR China*
† Author Contributions: These authors contributed equally to this work (joint first authors).
* Corresponding author: Kai Li, Tel: +86–0871–65920507; Fax: +86–0871–65920507; E–mail: likaikmust@163.com

Supplementary materials

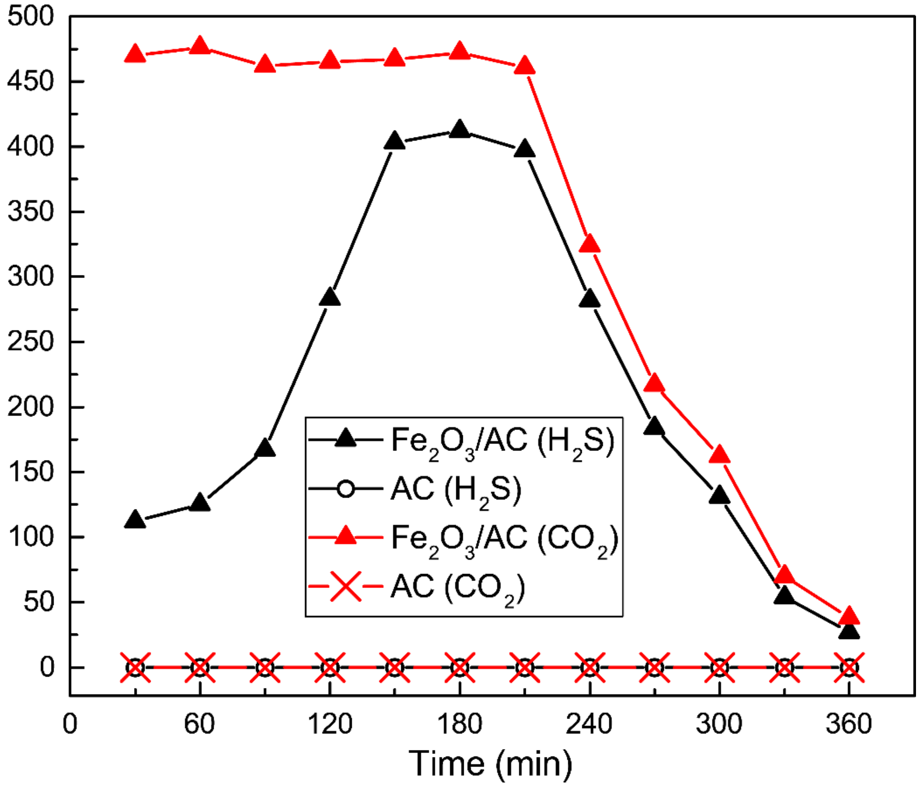

Figure S1. The effluent H_2S and CO_2 content over Fe_2O_3/AC and pure AC

(Experimental conditions: 15 ppm CS_2; 500 ppm COS; GHSV = 10000 h^{-1}; reaction temperature: 70 °C; RH = 49%; O_2 = 0%; inlet H_2S = 0%)

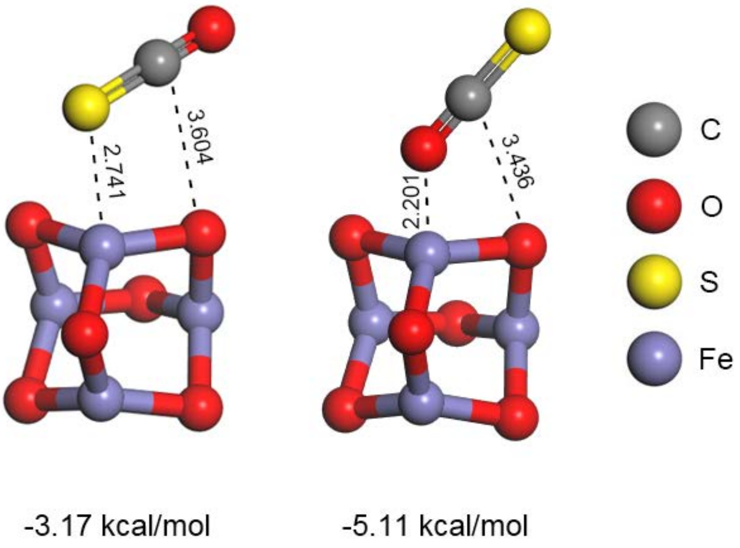

Figure S2. Position of COS adsorbed over Fe_2O_3 (Adsorption energy, kcal/mol)

Table S1. Imaginary frequencies of the transition states, bonds corresponding to relative normal vibrations and bond-lengths for COS hydrolysis

Transition States	Imaginary frequency (cm^{-1})	Bonds corresponding to relative normal vibrations	Bond-lengths (Å)
TS1	–906.47	H3–O6	2.192
TS2	–1528.43	H3–S4	1.679
TS3	–753.33	H3–S4	1.937
TS4	–1643.95	H2–S4	1.717

Table S2. Imaginary frequencies of the transition states, bonds corresponding to relative normal vibrations and bond-lengths for step 1 of CS_2 hydrolysis

Transition States	Imaginary frequency (cm^{-1})	Bonds corresponding to relative normal vibrations	Bond-lengths (Å)
TS5	–703.92	H14–S16	1.682
TS6	–1045.81	H15–S18	3.009
TS7	–1019.70	H15–S16	1.609
TS8	–1546.07	H15–S16	1.641

Table S3. Imaginary frequencies of the transition states, bonds corresponding to relative normal vibrations and bond-lengths for step 2 of CS_2 hydrolysis

Transition States	Imaginary frequency (cm^{-1})	Bonds corresponding to relative normal vibrations	Bond-lengths (Å)
TS9	–1624.81	H23–O13	1.292
TS10	–1325.24	H23–S18	2.503
TS11	–1172.61	H23–S18	1.556
TS12	–1495.37	H22–S18	1.707

Table S4. Reaction energy and energy barriers of different reaction channels

Reaction channels	Reaction energy (kcal/mol)	Reaction steps	Energy barrier (kcal/mol)
Channel I	-10.16	IM1-TS1-IM2	133.24
		IM2-TS2-IM3	46.75
		IM3-TS4-IM4	60.19
Channel II	-10.16	IM1-TS3-IM3	38.49
		IM3-TS4-IM4	60.19

Channel III	5.67	IM4-TS5-IM5	97.21
		IM5-TS6-IM6	36.76
		IM6-TS7-IM7	46.66
Channel IV	5.67	IM4-TS5-IM5	97.21
		IM5-TS8-IM7	48.57
Channel V	3.49	IM7-TS9-IM8	80.67
		IM8-TS10-IM9	97.76
		IM9-TS12-Product	43.71
Channel VI	3.49	IM7-TS11-IM9	66.17
		IM9-TS12-Product	43.71

Table S5. Surface reaction maximum energy barriers and gas-phase reaction maximum energy barriers of different reaction channels

Reaction channels	Surface reaction maximum energy barriers (kcal/mol)	Gas-phase reaction maximum energy barriers (kcal/mol)
Channel I	133.24	208.15
Channel II	60.19	159.74
Channel III	97.21 (46.66*)	182.71 (122.43*)
Channel IV	97.21 (48.57*)	182.71 (146.35*)
Channel V	97.76	217.39
Channel VI	66.17	163.25

Notes: * represent the second maximum energy barriers when first maximum energy barrier was same.
